# DKK2 blockage-mediated immunotherapy enhances anti-angiogenic therapy of *Kras* mutated colorectal cancer

**DOI:** 10.1016/j.biopha.2020.110229

**Published:** 2020-05-20

**Authors:** Jiajia Hu, Zhengting Wang, Zhengxi Chen, Ao Li, Jing Sun, Minhua Zheng, Jibo Wu, Tianli Shen, Ju Qiao, Li Lin, Biao Li, Dianqing Wu, Qian Xiao

**Affiliations:** a Department of Nuclear Medicine, Ruijin Hospital, Shanghai Jiao Tong University School of Medicine, Shanghai, China; b Department of Pharmacology and Vascular Biology and Therapeutic Program, Yale School of Medicine, New Haven, CT, United States; c Department of Gastroenterology, Ruijin Hospital, Shanghai Jiao Tong University School of Medicine, Shanghai, China; d Department of Orthodontics, Shanghai Ninth People’s Hospital, School of Stomatology, Shanghai Key Laboratory of Stomatology, Shanghai Jiao Tong University, Shanghai, China; e Department of General Surgery, Ruijin Hospital, Shanghai Jiao Tong University School of Medicine, Shanghai, China; f Institute of Biochemistry and Cell Biology, Shanghai Institutes for Biological Sciences, Chinese Academy of Sciences, University of Chinese Academy of Sciences, Shanghai, China; g Department of General Surgery, First Affiliated Hospital of Xi’an Jiaotong University, Xi’an, Shaanxi Province, China; h Department of Mechanical and Industrial Engineering, Northeastern University, Boston, MA, United States

**Keywords:** DKK2, KRAS, APC, Anti-VEGFR, Immune activation, Tumor microenvironment, Therapeutic approaches

## Abstract

There are limited options for targeted therapies for colorectal cancer (CRC). Anti-EGFR therapy is limited to CRC without KRAS mutations. Even worse, most of CRC are refractory to currently immune checkpoint blockade. DKK2, which is upregulated in CRC, was recently found to suppress host immune responses, and its blockage effectively impeded tumor progression in benign genetic CRC models in our previous study. Here, our recent study demonstrated that in human CRC tumor samples expressing high levels of DKK2, DKK2 blockade caused stronger activation of tumor infiltrating CD8^+^ T cells in *ex vivo* culture. Intriguingly, we observed a correlation of high DKK2 expression with increased lymph node metastasis prevalence in these CRC patients as well. Furthermore, in a mouse genetic CRC model with mutations in APC and KRAS, which more closely mimics advanced human CRC, we confirmed the tumor inhibitory effect of DKK2 blockade, which significantly retarded tumor progression and extended survival, with increased immune effector cell activation and reduced angiogenesis. Based on this, we performed a combined administration of DKK2 blockade with sub-optimal anti-VEGFR treatment and observed a synergetic effect on suppressing tumor angiogenesis and progression, as well as extending survival, better than those of every single therapy. Thus, this study provides further evidence for the potential therapeutic application of DKK2 blockade in the clinical treatment of human CRC.

## 1. Introduction

Colorectal cancer (CRC) is the third most common cancer in women and men, and is the third leading cause of cancer death in the United States, accounting for approximately 9% of all cancer deaths [1]. Treatment for colon cancer is based largely on the stages of cancer, and the major therapies include surgery resection, radiation, chemotherapies, and targeted therapies. The targeted therapies for CRC include Bevacizumab (Avastin), which targets VEGF and blood vessel formation, and Cetuximab (Erbitux), which targets epidermal growth factor receptor) [1]. However, these anti-EGFR drugs don’t work in CRC that has mutations (defects) in the KRAS, NRAS or BRAF gene [1].

Tumor growth is often restricted due to their metabolic demands for oxygen and nutrients, which have limited diffusion. For further growth, tumors require increased blood vessel formation, *i.e*., angiogenesis. Angiogenesis is not only necessary for tumors to grow, but also contributes to the spread of bloodborne metastases [2–4]. Vascular endothelial growth factor (VEGF) is a family of soluble protein growth factors, which consist of key mediators of angiogenesis and lymphangiogenesis in the context of tumor biology [5–8]. VEGF not only drives angiogenesis but also serves as a survival factor for endothelial cells and promotes the abnormal phenotype of blood vessels in tumors [7]. Anti-VEGF agents have been used widely in the treatment of many solid cancers [7,9]. However, major adverse events have been reported with the systemic administration of anti-VEGF monoclonal antibodies, including hypertension, impaired wound healing, hemorrhage and thrombosis, cardiac impairment, stroke, gastrointestinal perforations, and kidney disease [7,9]. These side effects reduce patient compliance and limit the application of anti-VEGF, particularly at the most effective dosage, on tumor therapy [7]. In colorectal cancer patients, even with bevacizumab combination with chemotherapy regimens, the median progression-free survival of metastatic colorectal cancer patients remains less than one year [7]. Therefore, improvement of the response rate and survival with new targeted agents remains an area of active investigation.

Recently, immunotherapy, particularly the immune checkpoint inhibitors, has provided a significant advance in cancer treatment. However, the currently approved checkpoint inhibitors including anti-PD1/PD-L1 and anti-CTLA4 are only efficacious for CRC with a high level of microsatellite instability (MSI-H), which are only accounted for less than 10 % CRC cases. Most CRC, which belongs to the microsatellite stable (MSI-S) group, are refractory to the treatment. We recently showed that DKK2, a canonical Wnt signaling pathway antagonist [10,11], which is upregulated in CRC, promoted tumor progression by suppressing the activation of immune effector cells [12]. We demonstrated that the DKK2 blockade with the anti-DKK2 antibody 5F8 was effective in suppressing tumor progression using a syngeneic CRC model grafted with mouse colon cancer cell MC38 and a genetic benign CRC model using the Apc^Min/+^ mice [12,13]. The gradual accumulation of genetic alterations in driver genes, such as APC, KRAS, SMAD4, TP53, and *PIK3A*, underlie the development and malignant progression of human colorectal cancers [14–16]. In this study, we revealed that enhanced cytotoxic T cell response of anti-DKK2 antibody 5F8 treatment is dependent on DKK2 expression in human colorectal cancer samples. Intriguingly, we observed a correlation between high DKK2 expression and increased lymph node metastasis prevalence in these CRC patients as well. Furthermore, we tested the effect of the DKK2 blockade on a mouse genetic CRC model that more closely mimics advanced human CRC. We found that the anti-DKK2 antibody 5F8 inhibited tumor progression in an advanced colon cancer model by modifying tumor immune microenvironment. Besides the regulation of DKK2 on cytotoxic immune cells, we also observed that 5F8 antibody treatment impaired tumor vasculature at the late stages of tumor progression.

A growing number of pre-clinical studies on combination therapies targeting both immune escape and tumor vascularization were proved to have promising effects [4,17–20]. Those preclinical studies could help to identify the most promising candidates for clinical trials and offer new insights into the biological mechanisms that underlie drug synergies. Thus, we also investigated the combination effect of DKK2 blockage on VEGF-A/VEGFR blockade-mediated suppression of tumor progression. Interestingly, we found a synergetic effect on suppressing tumor progression, as well as extending survival, by a combination administration of DKK2 blockade with a suboptimal dose of anti-VEGFR *via* activation of CD8^+^ T/NK cells and impaired tumor angiogenesis.

## 2. Materials and methods

### 2.1. Antibodies

mouse CD4–PE (eBioscience, 12-0042-82), mouse NK1.1–allophycocyanin (BioLegend, 108,710), mouse CD8a–PE–cyanine 7 (eBioscience, 25-0081-82), mouse CD69–PE (Biolegend, 104508), human/mouse granzyme B–FITC (BioLegend, 515403), mouse CD314 (NKG2D)-PE-cyanine 7 (eBioscience, 25-5882-81), mouse IFN-γ–PE (eBioscience, 12-7311-81), mouse CD45-eFluor 450 (eBioscience, 48-0459-41), mouse CD107a-V450 (BD, 560648), mouse CD8a-allophycocyanin (eBioscience, 17-0081-81), mouse CD279 (PD-1)-PE (BioLegend, 135205), mouse CD19–PE–cyanine 7 (eBioscience, 25-0193-81), Ki67 (Abcam, ab15580), cleaved caspase-3 (Asp175) (CST, 9661S), CD31 (Abcam ab, 28364), FITC-labeled AffiniPure F(ab)2 fragment donkey anti-mouse IgG (H + L) (Jackson Lab, 715-096-151), and Alexa Fluor 647-labeled AffiniPure F(ab)2 fragment goat anti-rabbit IgG (H + L) (Jackson Lab, 111-606-045). Mouse monoclonal antibody (mAb) to DKK2 (5F8) was generated by standard hybridoma technology through immunization of mice with a synthetic peptide (KLNSIKSSLGGETPGC) of human DKK2 at AbMax (Beijing, China). Therapeutic antibodies to VEGFR were Clone DC101 (BioXcell, BE0060) with rat IgG1 isotype control (BioXcell, BE0091) as the control IgG.

### 2.2. Human colon tumor sample study

Sixteen patients with CRC, including 6 women and 10 men, were enrolled and pathologically diagnosed with colorectal adenocarcinoma at Ruijin Hospital, Shanghai Jiao Tong University School of Medicine from March 2018 to February 2019. Written informed consent was provided by all patients. This study protocol was following the approved guidelines and was approved by the Human Ethics Committee and the Research Ethics Committee of Ruijin Hospital. Fresh tumor and adjacent normal tissue samples (at least 2 cm from matched tumor tissues) were surgically resected from the above-described patients. Their ages ranged from 37 to 87 with a median of 63. None of them was treated with chemotherapy or radiation before tumor resection. The stages of these patients were classified according to the guidance of the AJCC version. Among these patients, one was diagnosed at stage I, seven at stage II, and eight at stage III. Among these patients, 8 had positive lymph nodes. None of those patients had distal metastasis, as evidenced by the enhanced computerized tomography (CT) results for abdomen, chest and pelvic areas before surgery. The available clinical characteristics are summarized in Supplementary Table 1.

### 2.3. Ex vivo cell culture

Fresh tumors and adjacent normal tissues were collected from surgical specimens after microscopical examination of the tissue by a pathologist. All tissues were washed by washing buffer (DMEM containing 10 % FBS and 65 mM DTT) in a shaker with the speed of 200 r/m at 37 °C for 15 min, and then were washed by cold 1*PBS thrice to remove DTT. Tissues were cut into small pieces (approximately 1 mm^3^) in the RPMI-1640 medium with 10 % fetal bovine serum, then were divided into 2 parts. One was incubated with anti-DKK2 antibody (40 μg/mL) and the other with control IgG, in the incubator at 37 °C for 18 h. After that, the tissue suspensions were enzymatically digested by Collagenase VIII at the concentration of 1 μg/mL in the incubator at 37 °C for one hour. The dissociated cells were subsequently passed through a 70-μm cell-strainer and centrifuged at 500*g* for 5 min. After the supernatant was removed, the pelleted cells were suspended in red blood cell lysis buffer and incubated on ice for 2 min to lyse red blood cells. After washing twice with cold 1*PBS, the cell pellets were re-suspended in sorting buffer (1*PBS supplemented with 2% FBS) and passed through a 40-μm cell-strainer. Lymphocytes were isolated by Ficoll density gradient technique according to standard protocol. Cells were re-suspended in sorting buffer for later FACS analysis.

### 2.4. In situ hybridization

*In situ* hybridization detection of DKK2 mRNA was carried out using the following reagents acquired from Advanced Cell Diagnostics, INC based on provided protocol: RNAscope® Target Retrieval Reagents (Cat#322000), RNAscope® Pretreat Reagents- H_2_0_2_ and ProteasePlus (Cat# 322330), RNAscope® 2.5 HD Detection Reagent- RED (Cat#322360), RNAscope® Wash Buffer Reagents (Cat#310091), BioCare EcoMount (Cat#320409), ImmECatdge™Hydrophobic Barrier Pen (Cat#310018) and the mouse Dkk2probe (#404841).

### 2.5. Mice

Kras^LSL-G12D^ (B6.129S4-Krastm4Tyj/J), and Apc^fl/fl^ (C57BL/6-Apctm1Tyj/J) mice [21,22] were acquired from the Jackson Laboratory. Wild-type C57BL/6 mice were purchased from Envigo (Harlan). Mice were housed in specific-pathogen-free conditions and cared for following US National Institutes of Health guidelines, and all procedures were approved by the Yale University Animal Care and Use Committee.

### 2.6. Mouse model of colon cancer

Kras^LSL-G12D^ (B6.129S4-Krastm4Tyj/J), and Apc^fl/fl^ (C57BL/6-Apctm1Tyj/J) mice were backcrossed to *C57BL/6* background 3 generations, then Kras^LSL-G12D^ mice were crossed with Apc^fl/fl^ mice. For adenoviral infection of the colonic epithelium [23], mice were fasted overnight and anesthetized using 2% isoflurane. The distal colon was washed with PBS *via* the I.V. catheter through the anus. Then, 100 μL trypsin was injected into the colon for 10–15 min. The lining of the distal colon was then mechanically abraded using a small caliber brush. After washing with PBS, 10^9^ pfu of adenovirus in 100 μL PBS was injected into the colon for 30 min through the anus. Four weeks later, Kras^LSL-G12D^; Apc^fl/fl^ mice were randomly divided into two groups (17 each) for intraperitoneal injection with either control IgG antibody (10 mg/kg) or anti-DKK2 antibody (10 mg/kg) every week for another 6 weeks. 6 pairs of mice were used for flow cytometry analysis, 6 pairs of mice were used for gross inspection and histopathological examination, and rest 5 pairs of mice were used for survival study. Mice were housed in specific-pathogen-free conditions and cared for following US National Institutes of Health guidelines, and all procedures were approved by the Yale University Animal Care and Use Committee.

### 2.7. Tumor syngeneic graft

MC38 cell line was purchased from Kerafast (Cat#ENH204-FP). MC38 colon carcinoma cells (0.5 × 10^6^) were mixed with BD Matrigel (Matrix Growth Factor Reduced) (BD, 354230) in 100 μL and injected subcutaneously (s.c.) into the right flanks of the backs of female C57BL/6 mice (8–10 weeks old). Tumor growth was measured with calipers, and size was expressed as one-half of the product of perpendicular length and square width in cubic millimeters. For antibody treatment, control IgG, anti-DKK2 (5F8) and anti-VEGFR antibodies were diluted in PBS, and 100 μL was injected intraperitoneally (i.p.) at the intervals indicated in the figures. For survival tests, mice were euthanized when the tumor size exceeded 1500 mm^3^.

### 2.8. Histology

Murine organs were fixed in phosphate-buffered 4% formaldehyde and embedded in paraffin. 5-μm thick sections were stained with hematoxylin and eosin (H&E). Immunohistochemistry on 5-μm paraffin sections using antibodies against Ki67 (Abcam, ab15580), cleaved caspase-3 (Asp175) (CST, 9661S), CD31 (Abcam ab, 28364), was performed as described [12,24,25].

### 2.9. Flow cytometry

Cells in single-cell suspension were fixed with 2% PFA (Santa-Cruz sc-281692). After washing with a Flow Cytometry Staining Buffer (eBioscience 00-4222-26), cells were stained with antibodies for cell surface marker for 1 h on ice in the dark. For staining of intracellular proteins, the cells were washed and resuspended in the Permeabilization Buffer (BD 554723) and stained by antibodies in the Permeabilization Buffer for 1 h on ice in the dark. The cells were then pelleted and resuspended in the Flow Cytometry Staining Buffer for flow cytometry analysis [26,27].

### 2.10. Statistical analysis and study design

Minimal group sizes for tumor progression studies were determined *via* power calculations with the DSS Researcher’s Toolkit with an α of 0.05 and power of 0.8. Animals were grouped unblinded, but randomized, and investigators were blinded for the qualification experiments. No samples or animals were excluded from the analysis. Assumptions concerning the data including normal distribution and similar variation between experimental groups were examined for appropriateness before statistical tests were conducted. Comparisons between two groups were performed by unpaired, two-tailed t-tests. Comparisons between more than two groups were performed by oneway ANOVA, whereas comparisons with two or more independent variable factors were performed by two-way ANOVA followed by Bonferroni’s post hoc correction using Prism 6.0 software (GraphPad). Statistical tests were done with biological replicates. P < 0.05 was considered statistically significant.

## 3. Results

### 3.1. DKK2 blockade-induced enhancement of cytotoxic T cell responses are correlated with DKK2 expression in human colorectal cancer

**Fig. 1.**
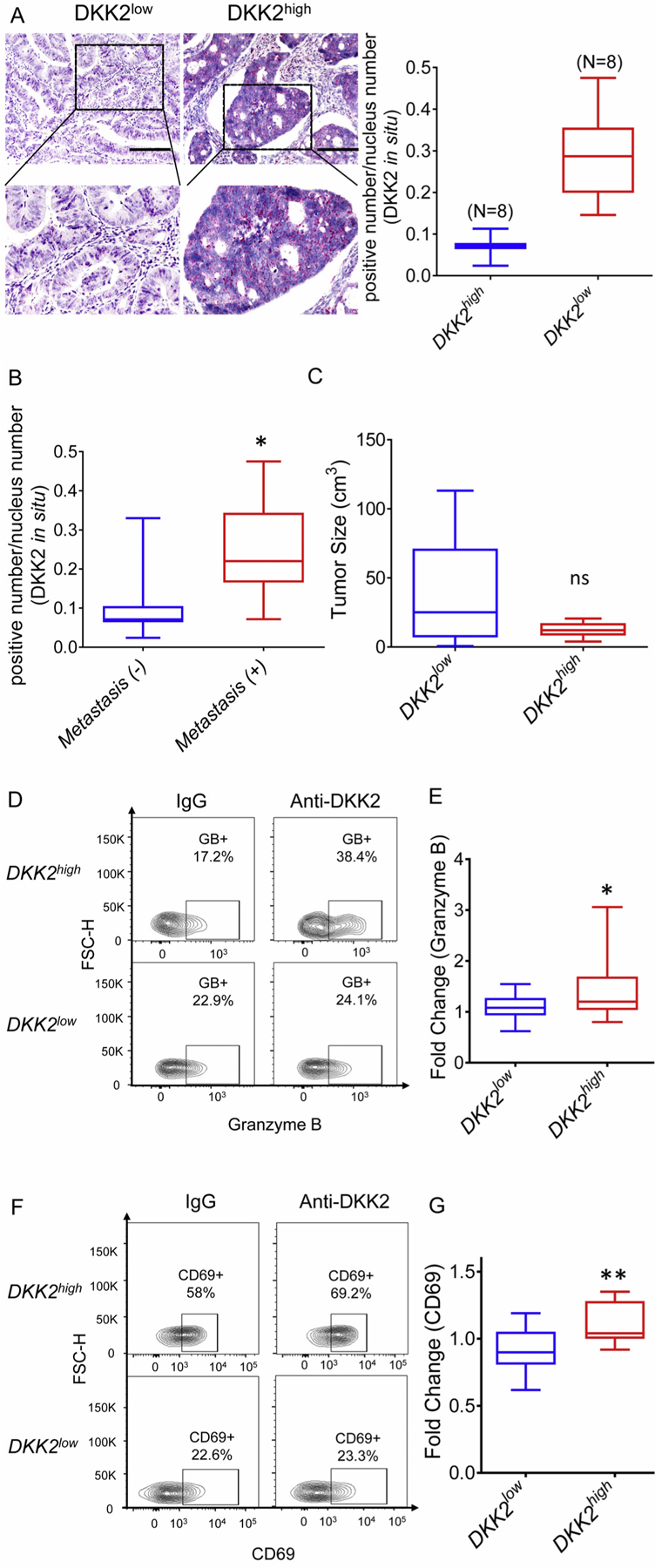
Enhanced cytotoxic response induced by anti-DKK2 treatment is dependent on Dkk2 expression in human colorectal cancer. (A) DKK2 mRNA in human intestinal tumor sections was detected by *in situ* hybridization. Based on DKK2 mRNA expression, the patients were divided into 2 groups: DKK2^high^ and DKK2^low^. Scale bars are 100 μm. (B) DKK2 expression in patients with or without juxtaintestinal lymph nodes metastasis. (C) Comparison of tumor size in DKK2^high^ and DKK2^low^ group. (D) Representative flow cytometry of granzyme B in Control IgG or anti-DKK2 (5F8) treatment of DKK2^high^ and DKK2^low^ group. (E) Fold change of granzyme B (anti-DKK2/Control IgG) in DKK2^high^ and DKK2^low^ group. (F) Representative flow cytometry of CD69 in Control IgG or anti-DKK2 (5F8) treatment of DKK2^high^ and DKK2^low^ group. (G) Fold change of CD69 (anti-DKK2/Control IgG) in DKK2^high^ and DKK2^low^ group (Two-sided Student’s t-test). (*P < 0.05; **P < 0.01; ***P < 0.001).

Our previous experiments demonstrated that DKK2 blockade with the anti-DKK2 antibody 5F8 was effective in suppressing tumor progression by enhancing immune effector CD8 + T cell activation, in a syngeneic CRC model grafted with mouse colon cancer cell MC38 and a genetic benign CRC model using the Apc^Min/+^ mice [12]. To verify whether DKK2 blockade activates infiltrating CD8^+^ T cells in human colorectal cancers, we collected 16 human colorectal tumor samples (Supplementary Table 1). One half of the surgically resected tumors were minced and cultured *ex vivo* in the presence of control IgG or 5F8 for 16 h, followed by an analysis of tumor infiltrating CD8^+^ T by flow cytometry. The other half of the tumors were sectioned for histological examination (Supplementary Table 1) and DKK2 expression analysis *via in situ* hybridization (Fig. 1A). Based on DKK2 mRNA levels in tumor sections, the samples were divided into the DKK2^high^ and DKK2^low^ groups (Fig. 1A). Anti-DKK2 treatment induced significant increases in GZMB and CD69 over control IgG treatment in the DKK2^high^, but not DKK2^low^ sample group (Fig. 1D–G), suggesting that DKK2 blockade activates infiltrating CD8^+^ T cells in human colorectal cancers that express high levels of DKK2. Interestingly, the patients with metastases in juxtaintestinal lymph nodes have higher expression levels of DKK2 in the original tumors than those without lymph node metastases (Fig. 1B), although there was no significant difference in the size of the original tumors between the DKK2^high^ and DKK2^low^ groups (Fig. 1C). This result suggests that DKK2 may play a role in colorectal cancer metastasis.

### 3.2. DKK2 blockade inhibits tumor progression in Kras^G12D/+^; Apc^fl/fl^

**Fig. 2.**
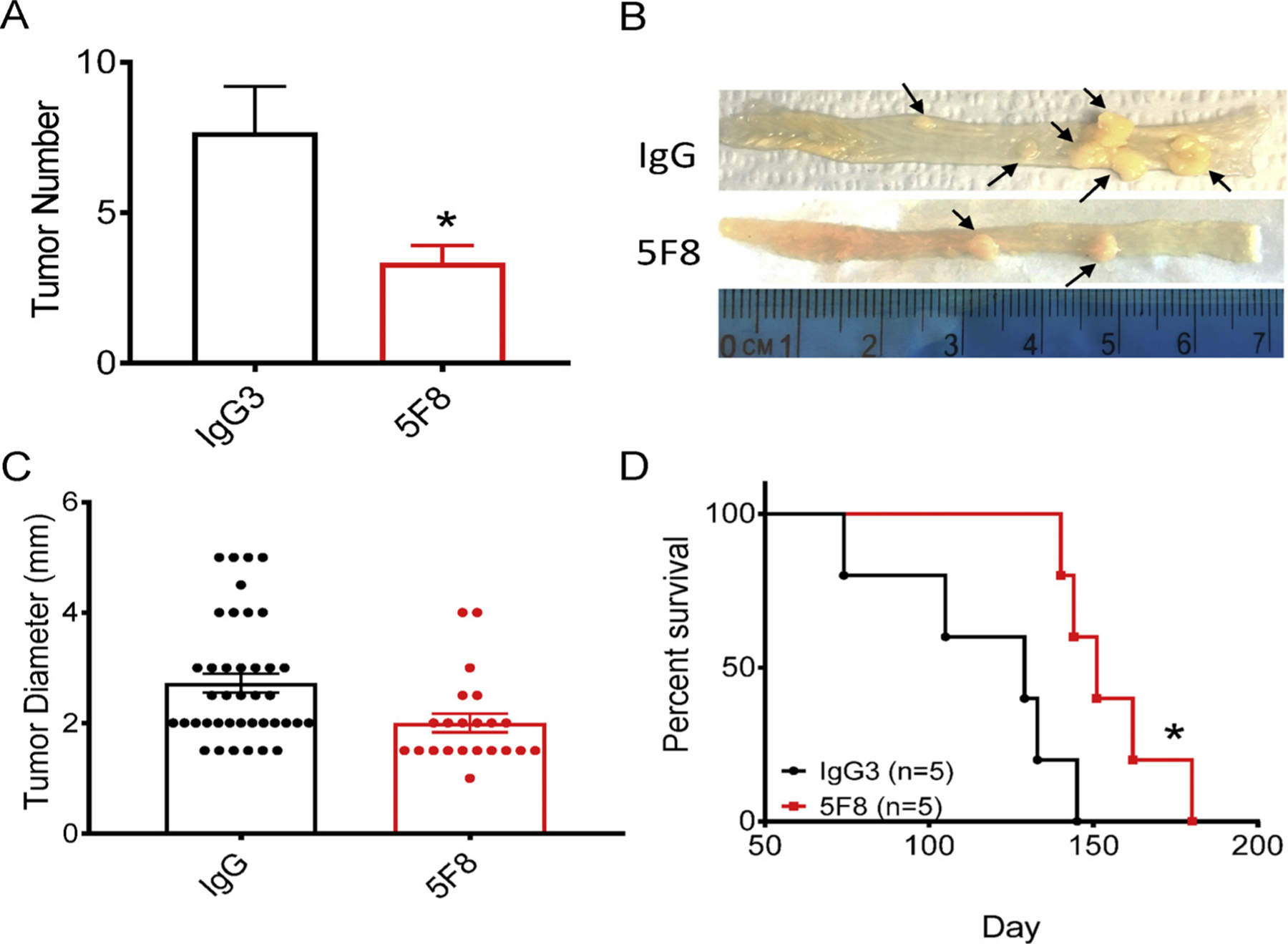
Anti-DKK2 antibody inhibited tumor progression in Kras^G12D/+^; Apc^fl/fl^ intestine tumor mouse model. (A–D) Kras^G12D/+^; Apc^fl/fl^ mice (n = 20) were administrated with the adeno-cre virus through the anus at age 10 weeks. When mice were 12-week-old, mice were divided into 2 groups randomly. Then mice were treated with anti-DKK2 (5F8) or an isotype antibody (IgG) (10 mg/kg i.p. once per week). (A–C) 5 pairs of mice were sacrificed at 16-week-old, tumors in the colon were counted and measured under a stereomicroscope after staining with methylene blue (*P* = 0.01; two-tailed Student’s *t*-test; *n* = 5). (D) 5 pairs of mice were monitored, and their survival was recorded (*P* = 0.02; two-sided Mantel-Cox log-rank test; *n* = 5). Data are presented as mean ± s.e.m.

**Fig. 3.**
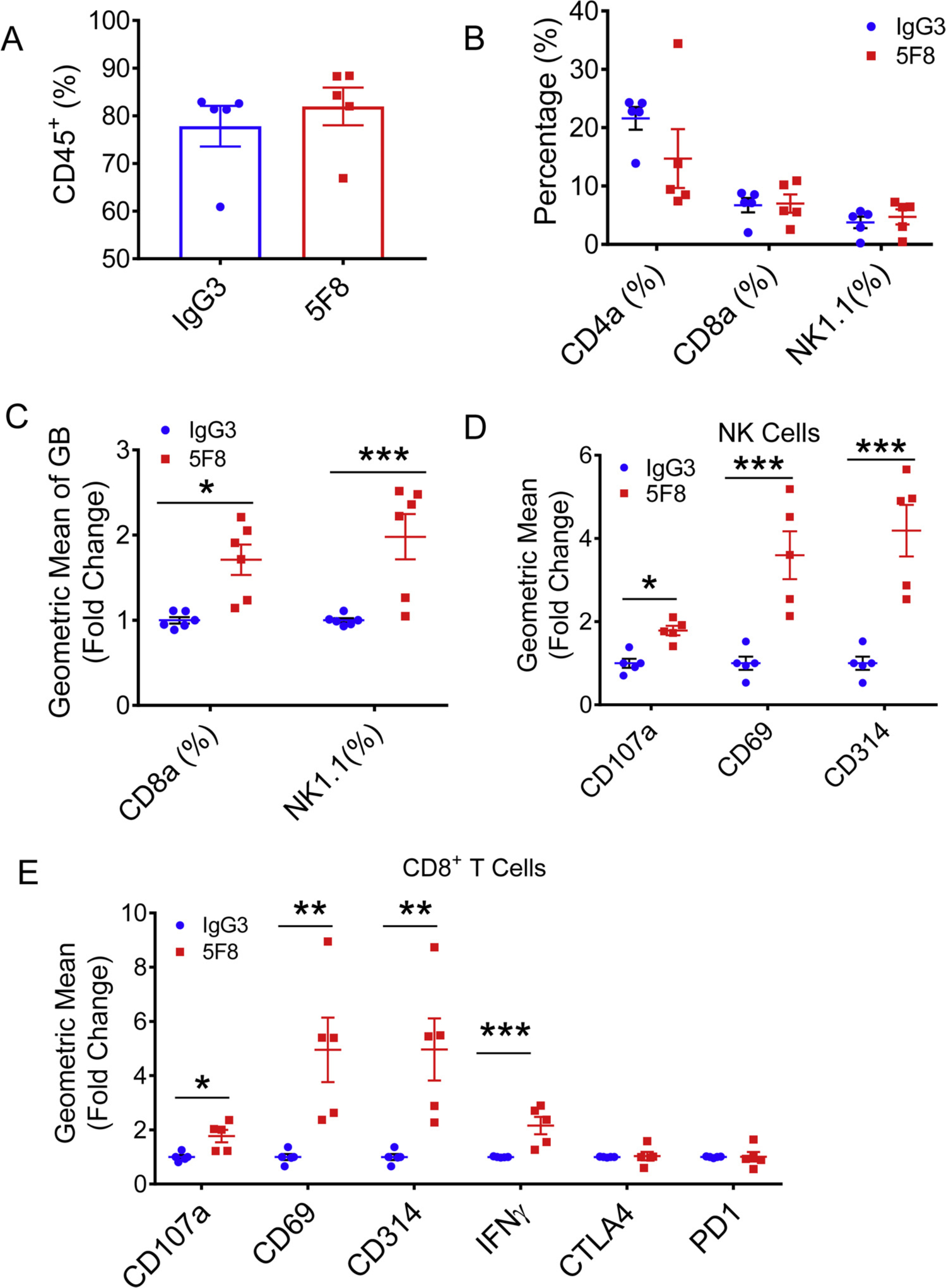
Administration of anti-DKK2 antibody enhanced cytotoxicity of NK and CD8^+^ T cells. (A–F) Flow cytometry analysis of tumor-in-filtrating lymphocytes in Kras^G12D/+^; Apc^fl/fl^ mice. Leukocytes from tumors from Kras^G12D/+^; Apc^fl/fl^ mice injected with of 5F8 or IgG (10 mg/Kg, i.p.) were prepared and analyzed by flow cytometry. (A) percentage of CD45 was shown. (B) CD4, CD8, NK were pre-gated from CD45^+^ population. (C) Granzyme B expression was measured in both CD8 and NK cells. (D) additional markers of tumor infiltrated NK1.1^+^ cells were described. (E) Flow cytometry analysis of additional markers of tumor infiltrated CD8^+^ T cells. Data are presented as means ± sem (Two-sided Student’s t-test; n = 5) (*P < 0.05; **P < 0.01; ***P < 0.001).

*mouse* To further evaluate the effect of the DKK2 blockade on a mouse model that more closely mimics advanced human CRC, we tested the anti-DKK2 antibody 5F8 in mice carrying the floxed Kras^G12D/+^ and Apc^fl/fl^ alleles. Upon administration of the Adeno-Cre virus to colons of these mice, tumors were formed at colons due to the lack of APC and expression of KRAS^G12D^, an activated KRAS mutant. Treatment of these mice with 5F8 significantly reduced tumor number and tumor size (Fig. 2A–C) and extended survival (Fig. 2D) compared with mice treated with an isotype control antibody. Flow cytometry analysis of tumor infiltrated leukocytes revealed that the DKK2 blockade did not significantly affect the infiltration of CD45^+^ (Fig. 3A, Supplementary Fig. 1A), CD4^+^ T cells, CD8^+^ T or natural killing (NK) cells (Fig. 3B) in the tumors. As same as in human colon cancer tissue, 5F8 treatment significantly increased the expression of Granzyme B (GZMB) in CD8^+^ T cells, as well as in NK cells (Fig. 3C). We also observed significant increases in levels of other activation markers of CD8^+^ T cells and NK cells, including CD69, CD107a, CD314, and IFN-γ in CD8^+^ T cells (Fig. 3E) and CD107a, CD69, and CD314 in NK cells (Fig. 3D). We did not observe any significant changes in levels of CTLA-4 or PD-1 in 5F8-treated CD8^+^ T cells compared with those from isotype IgG treated mice (Fig. 3E). These results suggest that the DKK2 blockade inhibits tumor progression in this advanced mouse CRC model accompanied by increased CD8^+^ T cell and NK cell activation.

### 3.3. DKK2 blockade inhibits tumor angiogenesis but increased immune effector cell activation

**Fig. 4.**
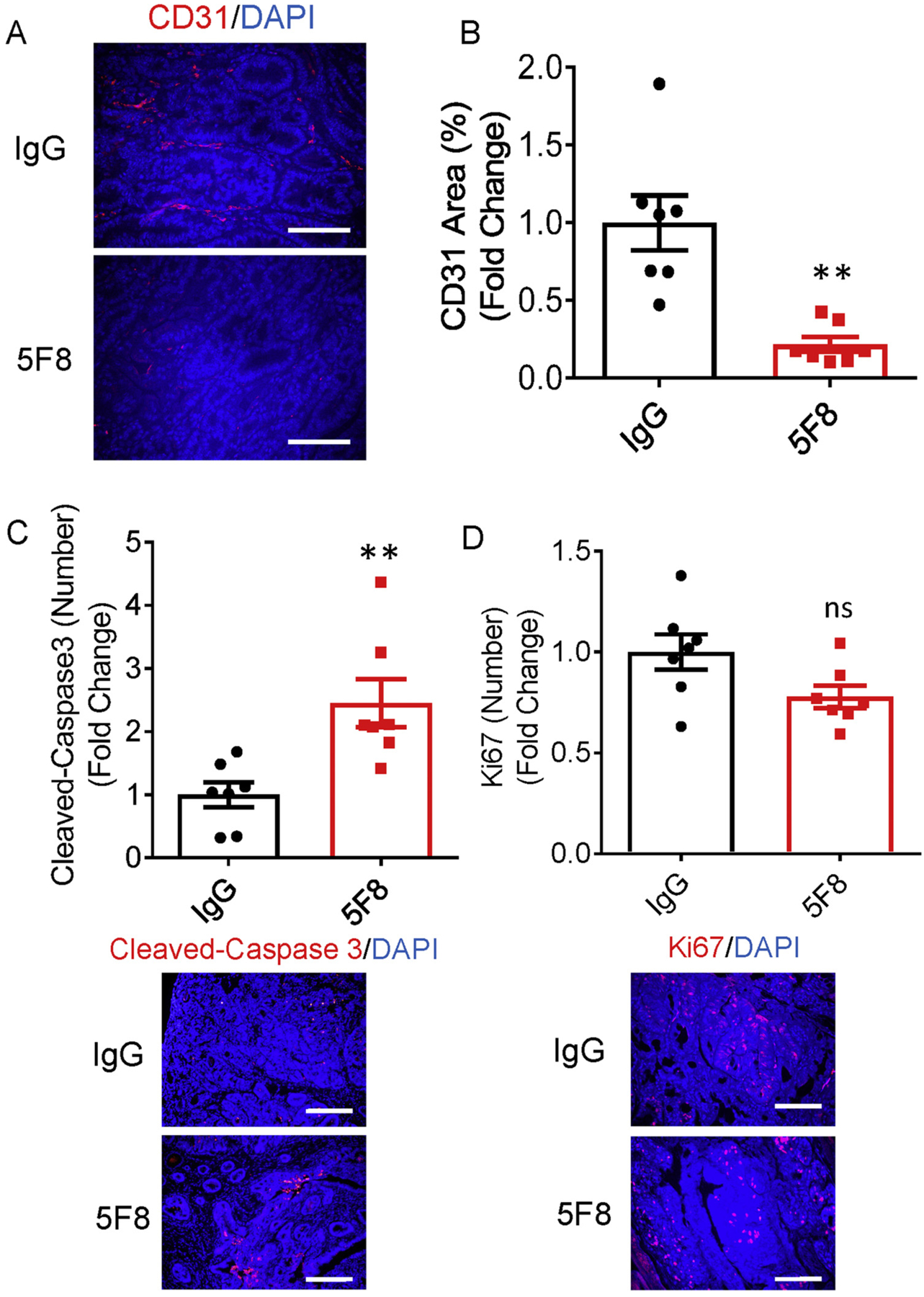
Anti-DKK2 treatment impaired tumor vasculature in Kras^G12D/+^; Apc^fl/fl^ mice. (A) Histological sections of colon tumors collected from Kras^G12D/+^; Apc^fl/fl^ mice injected with of 5F8 or IgG (10 mg/Kg, i.p. once per week) were stained with anti-CD31 antibody together with DAPI. Scale bars are 100 μm. Five independent sections per mouse were quantified from five mice per group. (B) Quantification of CD31 area (%) for each group. Data are presented as a means ± sem (Two-sided Student t-test). (*P < 0.05; **P < 0.01; ***P < 0.001). (C–D) Histological sections of colon tumors collected from Kras^G12D/+^; Apc^fl/fl^ mice injected with of 5F8 or IgG (10 mg/Kg, i.p. once per week) were stained with anti-Cleaved caspase-3 or Ki67 antibody together with DAPI. Scale bars are 100 μm. Five independent sections per mouse were quantified from five mice per group. Quantification of the number of Cleaved caspase-3 positive or Ki67 positive for each group. Data are presented as a means ± sem (Two-sided Student t-test). (*P < 0.05; **P < 0.01; ***P < 0.001).

The effects of DKK2 blockade on tumor proliferation, apoptosis, and neovascularization were assessed. Consistent with our previous study [12], DKK2 blockade increased the number of apoptotic cells (Fig. 4C) without significant effects on tumor cell proliferation (Fig. 4D). Unexpectedly, we found that there is a significant reduction of tumor neovascularization in the anti-DKK2 antibody treatment group (Fig. 4A–B). DKK2 blockade significantly increased IFNγ expression in CD8^+^ T (Fig. 3E) and NK cells [12]. Studies have demonstrated that IFNγ not only impedes tumor growth by acting directly on cancer cells [28,29], it also acts on the tumor stroma and tumor angiogenesis for effective rejection of large, established tumors [30–33]. The previous study also indicated that DKK2 directly promotes angiogenesis in rodent and human endothelial cells [34–37]. We were also able to reproduce the effects of DKK2 on angiogenesis using the tube formation assay *in vitro* and the Matrigel plug assay *in vivo*. This *in vitro* assay revealed that treatment of the cells with DKK2 protein profoundly enhanced tube formation in endothelial cells (Supplementary Fig. 2A–G). DKK2 also significantly increases angiogenesis compared with controls in the *in vivo* assay (Supplementary Fig. 3A–B). Taking these results together, DKK2 can promote angiogenesis, and anti-DKK2 antibody treatment may inhibit tumor angiogenesis directly and indirectly at the late stage of tumor progression.

### 3.4. Synergetic effect of combination treatment of anti-DKK2 and anti-VEGFR on the syngeneic graft tumor model

**Fig. 5.**
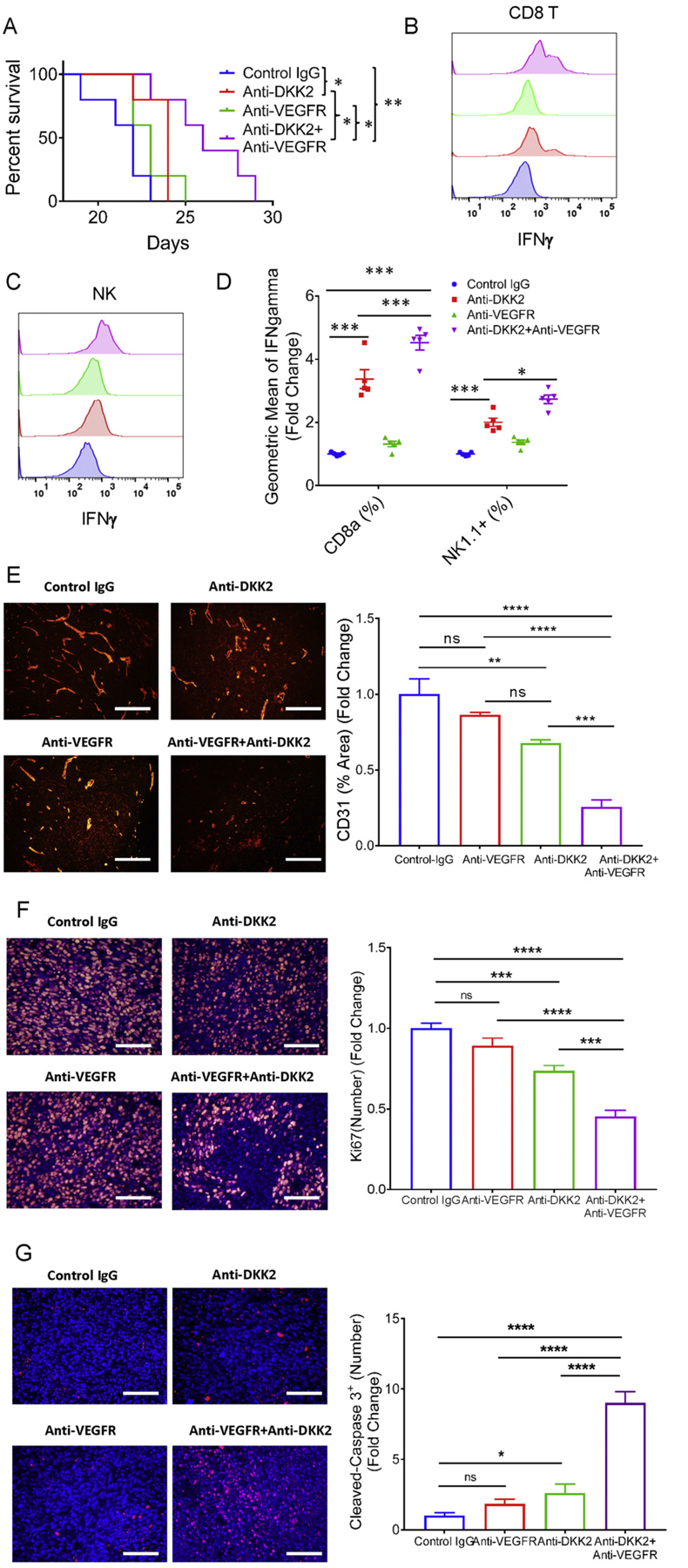
Combination treatment of anti-VEGF and anti-DKK2 further enhanced the activation of cytotoxic immune cells. (A) Augmented anti-tumor effects of DKK2 and VEGFR blockade combination in the MC38 tumor model. C57BL/6 mice were inoculated s.c. with MC38 cells. Treatment of IgG (12.5 mg/kg), anti-DKK2 (10 mg/kg)+IgG (2.5 mg/kg), anti-VEGFR (2.5 mg/kg)+IgG (10 mg/kg), and anti-DKK2 (10 mg/kg)+anti-VEGFR (2.5 mg/kg) in 100 μL was done at every 4 days starting Day 12. Survival was evaluated by the two-sided Log-rank (Mantel-Cox) multiple comparison test with Bonferroni correction (P = 0.005, IgG *vs* combo; P = 0.011, IgG *vs* anti-DKK2, P = 0.10 IgG *vs* anti-VEGFR; P = 0.046, combo *vs* anti-DKK2; P = 0.023, combo *vs* anti-VEGFR). (B–D) Effects of the antibody treatments on cytotoxic immune cells. C57BL/6 mice were inoculated s.c. with the MC38 cells. Treatments of anti-DKK2 (10 mg/kg, i.p) and/or anti-VEGFR (2.5 mg/kg, i.p) were done at Days 12, 15 and 18. Tumors were collected for flow cytometry analysis on Day 19. Flow data are presented as means ± sem (two-way Anova). (*P < 0.05; **P < 0.01; ***P < 0.001). (E) Histological sections of tumors from Figure 6A were stained with anti-CD31 antibody together with DAPI. Scale bars are 100 μm. Five independent sections per mouse were quantified from five mice per group. Quantification of CD31 area (%) for each group. Data are presented as means ± sem (Sidak’s multiple comparisons test). (*P < 0.05; **P < 0.01; ***P < 0.001). (F–G) Histological sections of tumors from Figure 6A were stained with anti-Ki67 or anti-cleaved caspase3 antibody together with DAPI. Scale bars are 100 μm. Five independent sections per mouse were quantified from five mice per group. Quantification of Ki67 number for each group. Data are presented as means ± sem (Bonferroni’s multiple comparisons test). (*P < 0.05; **P < 0.01; ***P < 0.001).

Given that anti-VEGF signaling is an approved therapy for human CRC, we tested if the combination therapy including VEGFR blockade and DKK2 blockade in the MC38 tumor syngeneic graft model could have a synergetic effect. Firstly, we applied previously reported an effective dosage of anti-VEGFR (10 mg/kg) [38–40] to the MC38 tumor syngeneic model in the presence or absence of 5F8 on Day 6 after tumor incubation. Both DKK2 blockade and VEGFR blockade showed significant tumor-suppressive effects, and the VEGFR blockade demonstrated a superior anti-tumor effect compared to the DKK2 blockade (Supplementary Fig. 4A–B). The combination treatment showed numeric, but not statistically significant, further retardation of tumor suppression compared to anti-VEGFR treatment alone (Supplementary Fig. 4A–B). Analysis of tumor sections revealed that the VEGFR blockade showed a strong inhibitory effect on neovascularization compared to the control IgG group or DKK2 blockade group. The combination treatment showed no further enhancement in the anti-angiogenesis effect (Supplementary Fig. 4C–D). From a clinical perspective, the purpose of combination therapy is to reduce the dose of a single therapeutic drug and thereby reduce the toxic side effects. We then tested a sub-optimal dose (2.5 mg/kg) of anti-VEGFR [38–40]. This dose of anti-VEGFR showed a smaller inhibitory effect on tumor progression (Supplementary Fig. 5A) than that of the full therapeutic dose but still had a tumor inhibitory effect that the tumor weight was lighter than that of the control IgG group at 19 days when tumors were collected for flow cytometry analyses (Supplementary Fig. 5B). However, this sub-optimal dose of anti-VEGFR did not provide significant long-term survival benefit over the control antibody (Fig. 5A & Supplementary Fig. 5C–D). DKK2 blockage showed a stronger effect on tumor suppression than this sub-optimal dose of anti-VEFGR (Supplementary Fig. 5A–B) and extended survival (Fig. 5A & Supplementary Fig. 5C–D). The combination yielded significant additional long-term survival benefit compared with individual blockade (Fig. 5A, Supplementary Fig. 5C–D), even though the combination did not exert a significantly further effect on tumor weight compared with DKK2 blockade alone at Day 19 (Supplementary Fig. 5A–B). Analysis of tumor-infiltrating lymphocytes showed that DKK2 blockades led to increased levels of GZMB in tumor-infiltrating CD8^+^ T cells and NK cells (Supplementary Fig. 5E–H). Importantly, the combination blockade resulted in the further increased level in IFNγ expression in these cells while DKK2 blockade alone increased the level of IFNγ in both CD8^+^ T cells and NK cells (Fig. 5B–D, Supplementary Fig. 1B). This effect of the combination of IFNγ expression suggests that VEGF-A/VEGFR plays an important role in the development of an immunosuppressive microenvironment involved in CD8^+^ T/NK cell dys-function. Although the sub-optimal dose of VEGFR blockade only showed a trend inhibitory effect on neovascularization and tumor proliferation, compared with the control IgG group (Fig. 5E–F), the combination treatment had a strong anti-angiogenesis effect (Fig. 5E). Consistently, the number of Ki67 positive cells was also decreased in the combo treatment group, compared to the control IgG group or single antibody treatment group (Fig. 5F). Further examination of cleaved caspase3 showed that the combo treatment group has a dramatic increased cleaved caspase3+ cells (Fig. 5G). Given these results, the DKK2 blockade can be used with anti-VEGFR therapy and even suboptimal doses of anti-VEGFR would provide additional benefits to the immunomodulatory effects of DKK2 blockade in treating colon tumors.

## 4. Discussion

In this study, we examined the effects of the DKK2 antibody on tumor progression and microenvironments in human CRC samples and mouse CRC models. We found that DKK2 blockade retarded tumor progression by increasing cytotoxic immune cell activation and suppressing tumor angiogenesis at the late tumor progression stage. We also found that the combination of anti-DKK2 can be used with anti-VEGFR in a mouse CRC model and notably DKK2-blockade can augment anti-tumor efficacy of sub-optimal dosage of anti-VEGFR by further reducing tumor angiogenesis and enhancing anti-tumor immunity.

In our previous study, we uncovered the function of DKK2 in promoting tumor progression by suppressing NK cell and CD8^+^ T cell activation in Apc^Min/+^ mouse intestinal tumor model [12]. It was not determined if anti-DKK2 blockage could also gain the similar tumor inhibitory effect in advanced colon tumors. With this study, we found that there is an enhanced cytotoxic T cell response after anti-DKK2 antibody treatment, which is dependent on DKK2 expression *via in vitro* human colorectal cancer tissue culture system (Fig. 1). Meanwhile, we showed that the DKK2 blockade has the same anti-tumor effects in the Kras^G12D/+^; Apc^fl/fl^ mice (Fig. 2). Interestingly, we observed that there was a correlation between lymph node metastasis and DKK2 expression in the original tumors (Fig. 1B). These findings are consistent with previous studies which demonstrated that DKK2 positively regulates tumor cell metastasis [34–36,41].

Tumors can grow only to a small size before their metabolic demands are restricted due to the diffusion limit of oxygen and nutrients [2]. To grow beyond this size, the tumor switches to an angiogenic phenotype and attracts blood vessels from the surrounding stroma [2,3,8]. This process is a prerequisite for the further outgrowth of the tumor. The previous study demonstrated that DKK2 has distinct functions of DKK1 [34]. DKK2 induction promoted angiogenesis in cultured human endothelial cells and in *in vivo* assays using mice, while DKK1 suppressed angiogenesis and was repressed upon induction of morphogenesis [34,36]. Those previous studies indicated that DKK2 directly promotes angiogenesis in rodent and human endothelial cells [34–37]. In this study, we also reproduced the pro-angiogenic effects of recombinant DKK2 proteins. On the other hand, studies also demonstrated that IFNγ not only impedes tumor growth by acting directly on cancer cells [28,29], it also acts on the tumor stroma and tumor angiogenesis for effective rejection of large, established tumors [30–33]. We have shown that DKK2 blockade significantly increased IFNγ expression in both CD8^+^ T and NK cells [12]. This suggests that the DKK2 blockade can also inhibit angiogenesis indirectly through its regulation on IFNγ. Thus, the marked anti-angiogenic effects observed in 5F8-treated mouse CRC models may be due to both direct and indirect effects DKK2 blockade on angiogenesis. Given that the DKK2 blockade had insignificant effects on tumor angiogenesis at the early stages of tumor progression [12], most of the anti-tumor effects of DKK2 blockade are likely the results of its effects on modulation of tumor immune microenvironments.

VEGF signaling plays an important role in tumor angiogenesis and cancer growth, researchers have made numerous attempts to decrease its expression and prevent tumor growth [7–9]. Recent research has shown that blood endothelial cells forming the tumor vessels can actively suppress the recruitment, adhesion, and activity of T cells [42–44]. Likewise, during tumorigenesis, the lymphatic vasculature undergoes dramatic remodeling that facilitates the metastatic spreading of cancer cells and immunosuppression [42,44,45]. More and more emerging evidence is highlighting the major role played by tumor-associated blood or lymphatic vasculature in thwarting immunosurveillance mechanisms and antitumor immunity [42–44]. This indicates that targeting of tumor vasculature might improve the efficacy of cancer immunotherapy. Considering that anti-DKK2 enhanced activation of cytotoxic immune cells, and VEGF-A/VEGFR was involved in the inhibition of dendritic cell maturation, accumulation of MDSC, and induction of T_reg_ cells, and CD8^+^ T cell activation/exhaustion [38,46–50], whether combination treatment with immune modulators may provide a better solution for Anti-VEGF therapy? Our study demonstrated that VEGFR blockage further increased the expression level of IFNγ in both CD8^+^ T cells and NK cells when it combined with immunomodulator anti-DKK2 antibody 5F8, compared to single antibody treatment. It is of note that the combination of anti-DKK2 antibody and VEGFR blockade induced a strong antitumor effect.

The dose of anti-VEGF agents matters [51–53]. Preclinical data indicate that high doses of anti-VEGF agents lead to increased deposition of the extracellular matrix that, together with hypoxia, can promote the infiltration of immunosuppressive and/or pro-tumor immune cells, such as monocytic and granulocytic MDSCs [54–56]. These results suggest that high-dose anti-VEGF therapy limits the benefits of chemotherapy in mouse models and patients with metastatic colorectal cancer (CRC) or advanced-stage NSCLC [54,57,58]. By contrast, the use of lower doses of anti-angiogenic agents, for instance, as low as one-quarter of the doses that induce anti-vascular effects has the potential to induce prolonged vessel normalization [59,60]. The results of two retrospective studies have shown that low-dose bevacizumab (< 3.6 mg/kg per week) in combination with chemoradiotherapy confers a greater survival benefit than high-dose bevacizumab in patients with glioblastoma [61–64]. Our study also has proved that the use of lower sub-optimal doses of the anti-VEGFR antibody, combined with the anti-DKK2 antibody, could significantly modify the immune response environment.

Our study provided a novel therapeutic approach combining VEGFR blockade and DKK2 blockade, which target the tumor vasculature and boosting immune cells’ killing capacity to help to overcome immunotherapy resistance. More work is needed to further elucidate the mechanism by which DKK2 regulates endothelial cells and its crosstalk with VEGF-A/VEGFR pathway during tumor progression. The potential combinatorial strategies using antiangiogenic and immunotherapy approaches also need to be further evaluated.

## 5. Conclusions

There are limited options for targeted therapies for colorectal cancer (CRC). Anti-EGFR therapy is limited to CRC without KRAS mutations. Even worse, most of CRC are refractory to currently immune checkpoint blockade. Our recent study demonstrated that in human CRC tumor samples expressing high levels of DKK2, DKK2 blockade caused stronger activation of tumor infiltrated CD8^+^ T cells in *ex vivo* culture. In a mouse genetic CRC model with mutations in APC and KRAS, which more closely mimics advanced human CRC, we confirmed the tumor inhibitory effect of DKK2 blockade, which significantly retarded tumor progression and extended survival, with increased immune effector cell activation and reduced angiogenesis. Based on this, our study provided a novel therapeutic approach combining VEGFR blockade and DKK2 blockade, which target the tumor vasculature and boosting immune cells’ killing capacity to help to overcome immunotherapy resistance. Thus, this study provides further evidence for the potential therapeutic application of DKK2 blockade in the clinical treatment of human CRC.

## Supplementary Material

Supp1

supp2
