# Supplementary material for: DKK2 blockage-mediated immunotherapy enhances anti-angiogenic therapy of Kras mutated colorectal cancer
Source: Biomed Pharmacother. Author manuscript; Available in PMC 2021 Jul 1. (PMC7523634; doi:10.1016/j.biopha.2020.110229)
Supplement: Supp1 [file NIHMS1628938-supplement-Supp1.docx]

**
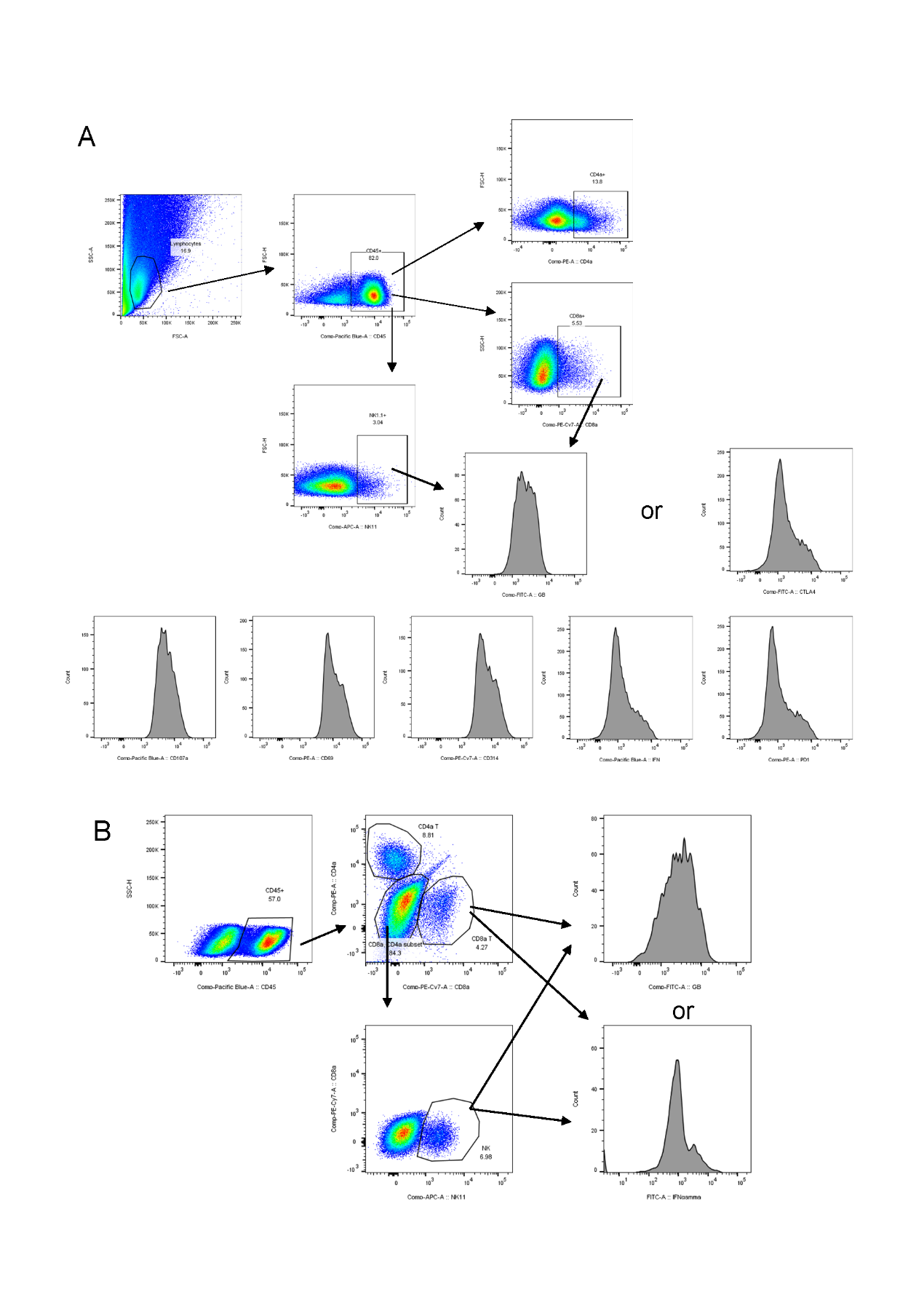
Supplementary Figure 1. Flow cytometry gating strategy.**

(A) Flow cytometry gating strategy for Figure 3. (B) Flow cytometry gating strategy for Figure 5.


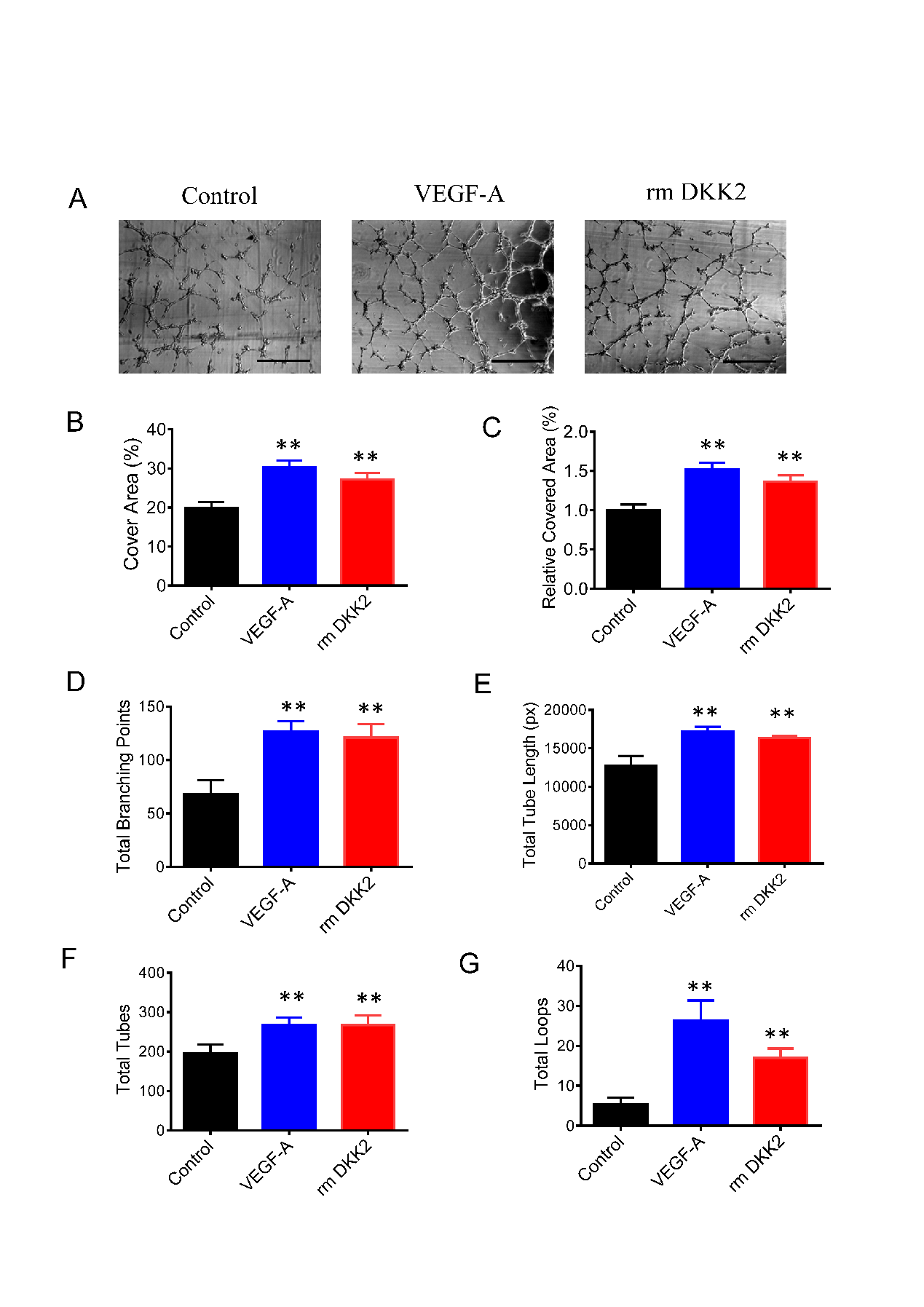


**Supplementary Figure 2. rm DKK2 promoted tube formation of endothelial cells**

(A-G) The primary cultured HUVECs were serum-starved for 24 hours and were exposed to PBS, VEGF-A, or DKK2 protein (200 ng/ml) for 48 hours in Matrigel based 2D-culture system. (A) microphotographs were taken, and representative endothelial tubes were shown. (B-G) key parameters of the tube formation network were quantified with online Wimasis Image Analysis website (https://mywim.wimasis.com/) (B) Cell covered area (%), (C) Relative Cell covered area (%), (D) Total Branching Points, (E) Total Tube Length, (F) Total Tubes, (G) Total Loops Data are mean ± SD; **p < 0.01 or ***p < 0.001.


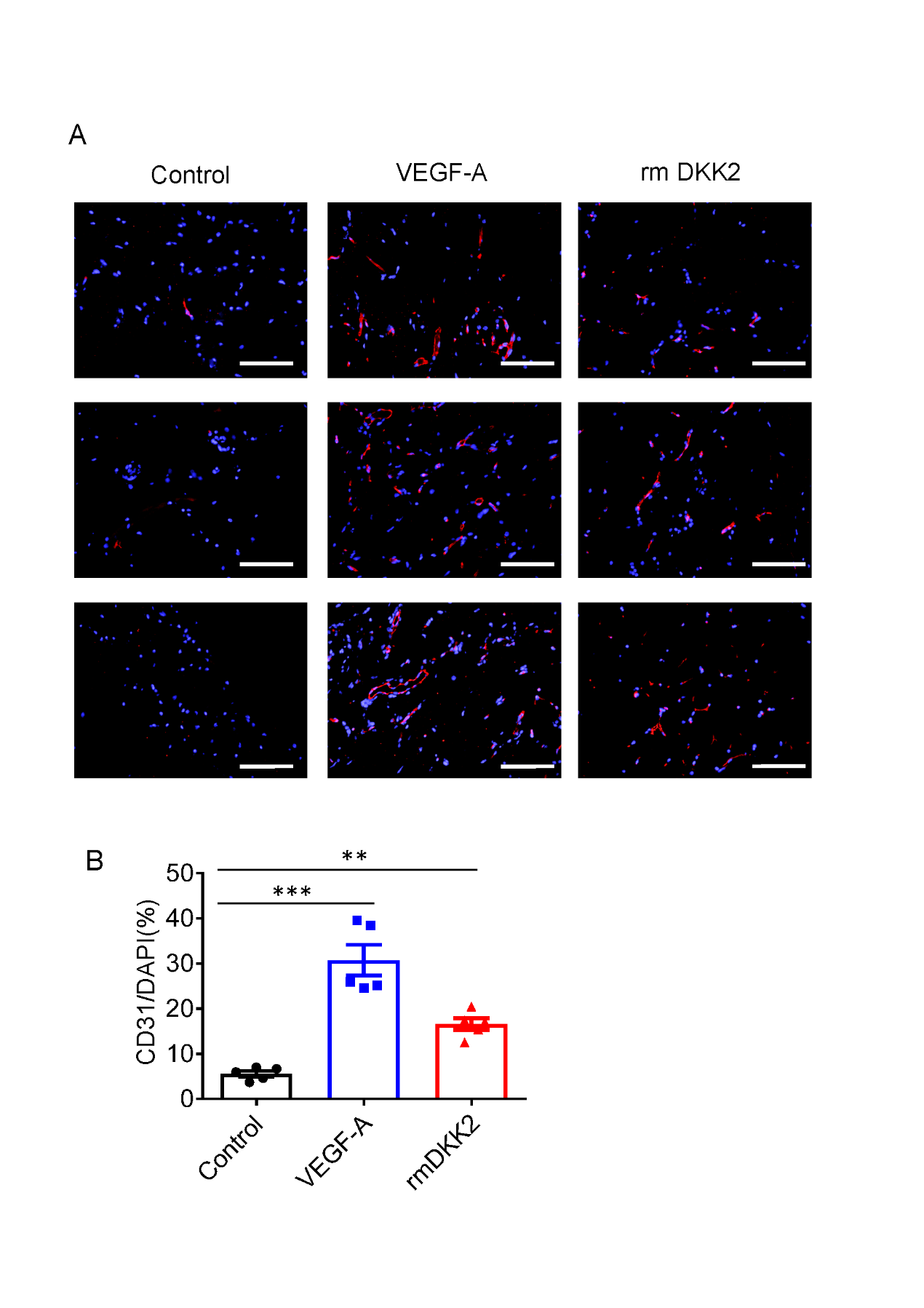


**Supplementary Figure 3. DKK2 protein induces angiogenesis *in vivo*.**

(A and B) Matrigel plugs treated with VEGF (400 ng) and DKK2 (1ug) were excised from mice 7 days after injection (*n* = 5 per group). (A) CD31 staining of slides from Matrigel plugs. Red, CD31 positive; blue, DAPI. Scale bars: 100 µm. (B) quantification of CD31 positive vs DAPI. Data represent mean ± SD. **p < 0.01 or ***P < 0.001.


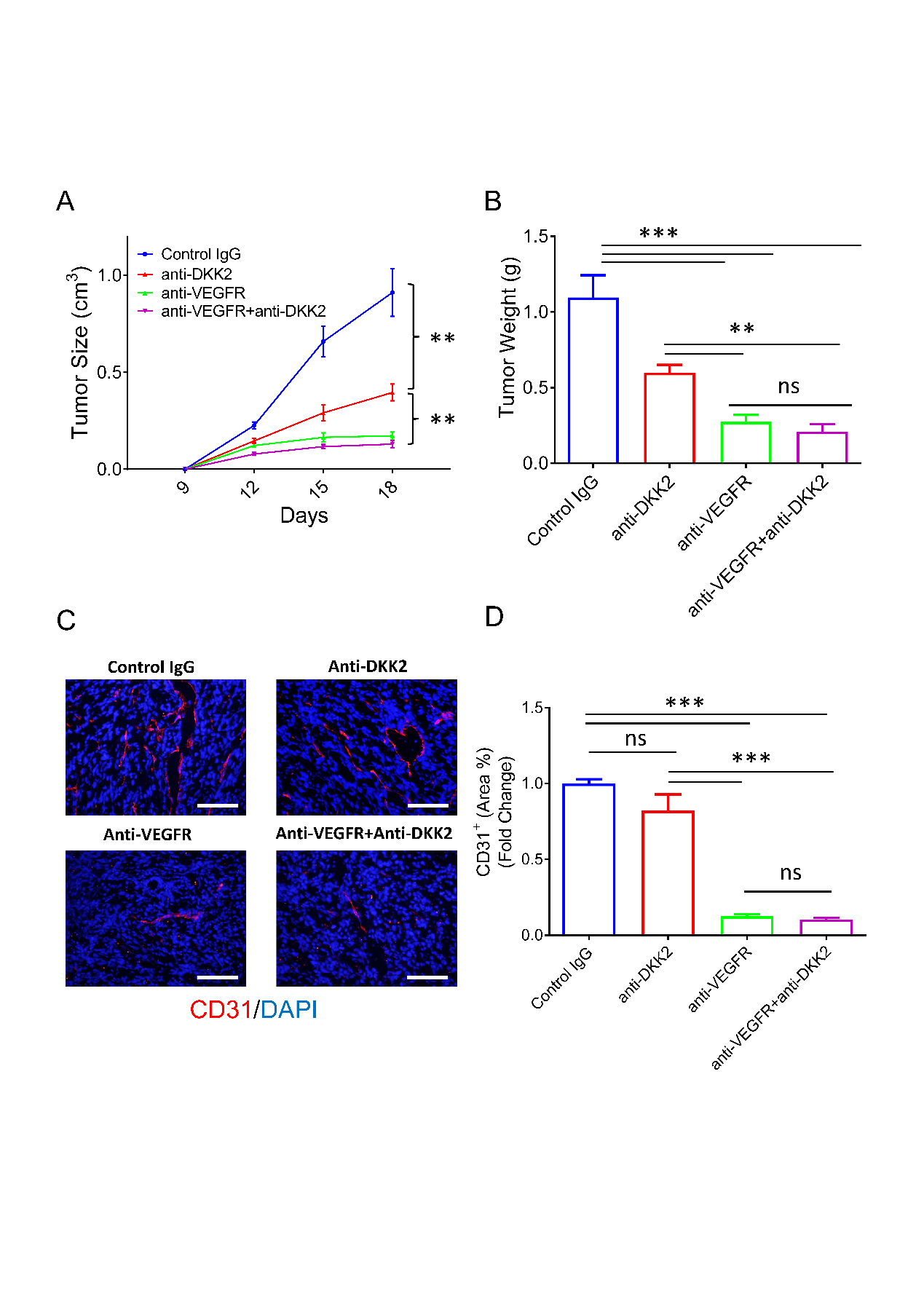


**Supplementary Figure 4. Combination treatment of anti-VEGFR at high dosage and anti-DKK2.**

(A) Tumor growth of the MC38 tumor model. C57BL/6 mice were inoculated s.c. with MC38 cells. Treatment of IgG (20 mg/kg), anti-DKK2 (10 mg/kg)+IgG (10 mg/kg), anti-VEGFR (10mg/kg)+IgG (10 mg/kg), and anti-DKK2 (10 mg/kg)+anti-VEGFR (10 mg/kg) in 100 µl was done at every 2 days starting Day 6. (B) Tumor weight at end of time point. (C) Histological sections of tumors from IgG (20 mg/kg), anti-DKK2 (10 mg/kg)+IgG (10 mg/kg), anti-VEGFR (10mg/kg)+IgG (10 mg/kg), and anti-DKK2 (10 mg/kg)+anti-VEGFR (10 mg/kg) were stained with anti-CD31 antibody together with DAPI. Scale bars are 100 µm. Five independent sections per mouse were quantified from five mice per group. (D) Quantification of CD31 area (%) for each group. Data are presented as means±sem (Two-sided Student t-test). (*P<0.05; **P<0.01; ***P<0.001).


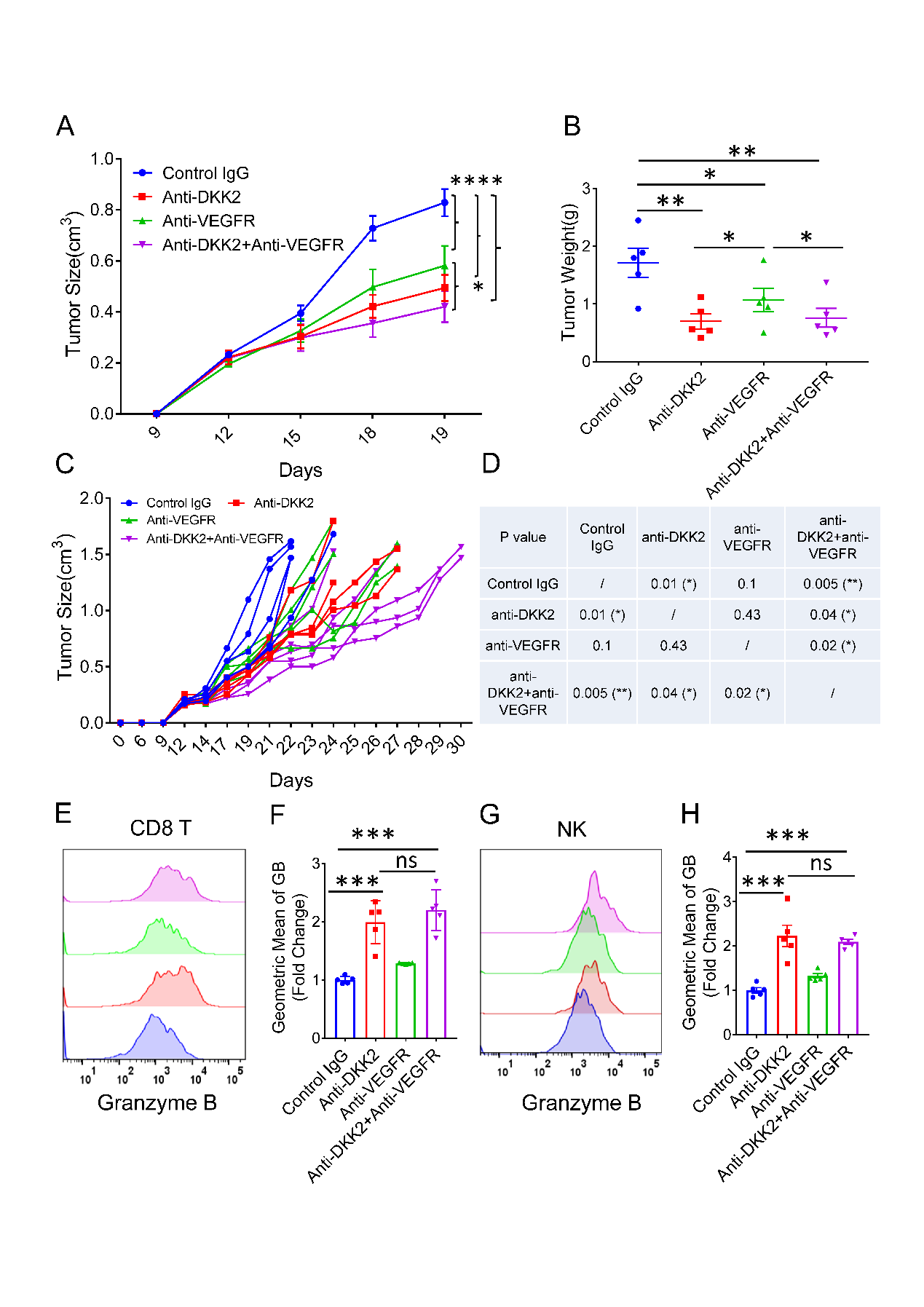


**Supplementary Figure 5. Augmented anti-tumor effects of DKK2 and VEGFR blockade combination in the MC38 tumor model.**

(A) C57BL/6 mice were inoculated s.c. with MC38 cells. Treatment of IgG (12.5 mg/kg), anti-DKK2 (10 mg/kg)+IgG (2.5 mg/kg), anti-VEGFR (2.5 mg/kg)+IgG (10 mg/kg), and anti-DKK2 (10 mg/kg)+anti-VEGFR (2.5 mg/kg) in 100 µl was done at every 4 days starting Day 12. Tumors were collected for FACS analysis at Day 19. (B) Tumor weight at end of time point for FACS analysis. (C) C57BL/6 mice were inoculated s.c. with MC38 cells. Treatment of IgG (12.5 mg/kg), anti-DKK2 (10 mg/kg)+IgG (2.5 mg/kg), anti-VEGFR (2.5 mg/kg)+IgG (10 mg/kg), and anti-DKK2 (10 mg/kg)+anti-VEGFR (2.5 mg/kg) in 100 µl was done at every 4 days starting Day 12. Survival was evaluated by the two-sided Log-rank (Mantel-Cox) multiple comparison test with Bonferroni correction. Survival for Figure 6A (IgG vs anti-DKK2; IgG vs anti-VEGFR; IgG vs combo; combo vs anti-DKK2; combo vs anti-VEGFR); (D) Single tumor growth for Figure 6A. (E-H) Effects of the antibody treatments on cytotoxic immune cells. C57BL/6 mice were inoculated s.c. with the MC38 cells. Treatments of anti-DKK2 (10 mg/kg, i.p) and/or anti-VEGFR (2.5 mg/kg, i.p) were done at Days 12, 15 and 18. Tumors were collected for flow cytometry analysis on Day 19. Flow data are presented as means±sem (two-way Anova). (*P<0.05; **P<0.01; ***P<0.001).
